# Cannulation Technique of Vascular Access in Hemodialysis and the Impact on the Arteriovenous Fistula Survival: Systematic Review and Meta-Analysis

**DOI:** 10.3390/jcm12185946

**Published:** 2023-09-13

**Authors:** Ricardo Peralta, Luís Sousa, António Filipe Cristovão

**Affiliations:** 1 Lisbon School of Nursing, University of Lisbon, 1600-096 Lisbon, Portugal; acristovao@esel.pt; 2 NephroCare Portugal, Fresenius Medical Care Portugal, 1750-233 Lisbon, Portugal; 3 School of Health Atlântica (ESSATLA), 2730-036 Oeiras, Portugal; luismmsousa@gmail.com; 4 Comprehensive Health Research Centre (CHRC), 7000-811 Evora, Portugal

**Keywords:** meta-analysis, end-stage renal disease, chronic kidney disease, hemodialysis, buttonhole, rope ladder, cannulation technique

## Abstract

Adequate cannulation technique (CT) methods and successful puncture are essential for hemodialysis (HD) and arteriovenous fistula (AVF) maintenance. This systematic review and meta-analysis was designed to identify which CT allows better AVF primary patency and lower rates of complications in HD patients. The search was carried out on the CINAHL, MEDLINE, Cochrane Library, and Joanna Briggs Institute Library databases to identify all randomized controlled trials (RCTs) and observational studies comparing clinical outcomes of buttonhole (BH) versus rope ladder cannulation (RL) from 2010 to 2022. The Risk-of-Bias (Rob 2) tool was used for RCTs and the ROBINS-I was used for non-randomized studies. RevMan 5.4 was used for the meta-analysis. A total of five RCTs, one quasi-randomized controlled trial, and six observational studies were included. When compared with RL cannulation, BH cannulation significantly increased bacteremia (RR, 2.76, 95% CI (1.14, 6.67), *p* = 0.02) but showed no differences in AVF primary patency (HR, 1.06, 95% CI (0.45, 4.21), *p* = 0.90). There was no thrombosis reduction (RR, 0.51, 95% CI (0.23, 1.14), *p* = 0.10) or intervention number reduction (RR, 0.93, 95% CI (0.49, 1.80), *p* = 0.84) with BH. Outcomes like pain, hematoma, and aneurism could not be merged due to a lack of data, reported as medians, as well as due to different definitions. The quality in general was poor and the heterogeneity among the studies prevented us from merging the outcomes.

## 1. Introduction

With the increase in the number of elderly patients, the exhaustion of vascular territory, and the emergence of diabetes as the primary cause of renal etiology, the establishment and preservation of suitable vascular access (VA) is essential for the successful treatment of patients with end-stage kidney disease (ESKD) on hemodialysis (HD) programs. A functioning VA is the lifeline [1,2] that allows patients the undergoing of HD such as renal replacement therapy, allowing their survival and the maintenance of an acceptable quality of life. Conversely, the preservation and maintenance of a complication-free VA remains the Achilles’ heel of this field [2,3]. Moreover, VA dysfunctions remain the major cause of comorbidities and hospitalizations [4,5,6] in ESKD patients. The selections of the most suitable cannulation technique (CT) and VA cannulation are the most important aspects in dialysis [7], and nurses have the responsibility for constantly updating their knowledge and skills in this area. Selection of the best technique is fundamental to the proper use of VA and allows effective treatment; the correct and appropriate choice of arteriovenous fistula (AVF) cannulation is the key to its preservation and the prevention of VA-related dysfunction [8]. The technique that has always been referenced and recommended as preferable for AVF cannulation is the rope ladder (RL) technique [9,10].

However, challenges have been identified in the use of this cannulation technique such as severe pain with an impact on treatment time [11,12] and an increased risk of hematomas [13]. However, nurses tend not to explore the entire length of the vessel due to the increased risk of infiltration, and even with a protocol for the use of the RL technique, they end up using area cannulation [14]. The area CT leads to a decreased vein wall and tissue thickening and consequent aneurysm formation, with the increased risk of vein wall rupture [8]. Despite this knowledge, it is the most commonly used technique in some European countries, being used in 65.8% (44% to 77%) of patients compared to 28.2% for RL and 6% for BH [15]. The BH technique has some limitations since it must be used exclusively in AVF and requires the cannulation to be performed by the same nurse until the tunnel is built, and it is time-consuming. Although some have reported the advantages of BH, others have reported increased risks of local infection and bacteremia [16,17], even after major re-education and asepsis technique campaigns [18].

In recent years, some studies have been published [17,19,20] that may contribute to clarifying which CT allows for greater fistula survival and fewer complications. Therefore, this study aims to identify which CT allows for greater AVF survival and a lower rate of complications and which CT causes less pain for patients who undergo regular hemodialysis.

## 2. Materials and Methods

### 2.1. Search Strategy

This systematic review and meta-analysis was conducted in accordance with Preferred Reporting Items for Systematic Reviews and Meta-Analysis (PRISMA) guidelines [21]. The protocol was registered in the PROSPERO database prior to commencement (registration number CRD42021237050).

**Figure 1:**
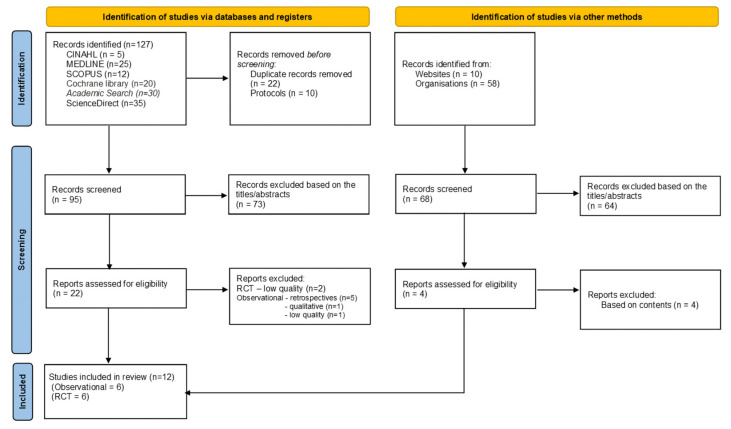
Study flow diagram.

We conducted searches on the EBSCO platform, accessing the Cumulative Index of Nursing and Allied Health Literature (CINAHL) and MEDLINE databases using the following Medical Subject Headings (MeSH) (2021) and strategy: ((MH “Dialysis”) OR (MH “Renal Dialysis”) OR (MH “Hemodialysis”) OR (MH “Kidney Failure Chronic”)) AND (((MH “Arteriovenous Fistula”) OR (MH “Catheterization”)) AND ((“Buttonhole”) OR (buttonhole) (OR constant site)) AND ((rope-ladder) OR (ropeladder) OR (rotating site))). We also considered other databases such as the Cochrane Library, ScienceDirect web, Joanna Briggs Institute Library Evidence-Based Practice Network (JBI), SCOPUS, ResearchGate, American Society of Nephrology (ASN), American Nephrology Nurses Association (ANNA), Sociedade Espanhola de Nefrologia (SEN), and Sociedade Brasileira de Nefrologia (SBN). We considered these databases because they publish randomized controlled trials (RCTs) and observational studies in the nephrology field. All the articles included in the review were available in full text. A flow chart is shown in Figure 1.

### 2.2. Inclusion and Exclusion Criteria

We included all RCTs and quasi-experimental and prospective observational studies published between January 2000 and January 2022 that satisfied the following criteria: studies that compare CTs and thus define the advantages and risks of each CT; primary and full studies, or abstracts that include one or some outcomes. We reviewed articles in English that enrolled adults aged 18 years and older who underwent hemodialysis using an autogenous AVF. Patients who underwent BH were considered the experimental group, and patients who underwent RL or other CT were considered the control group.

We excluded studies associating patient data from home hemodialysis with data from hospital or hemodialysis clinics and studies with incidents of patients on hemodialysis and qualitative studies.

### 2.3. Outcome Indicators

The primary outcome was the fistula primary patency, and according to Lee T. et al. [22], this was evaluated by the percentage of AVF in use from the study start to the time of the first clinical intervention for angioplasty or vascular surgery (unassisted patency).

The secondary outcome was considered fistula survival, for which failure was defined as AVF no longer used for successful HD. The numbers of interventions, thrombosis, bacteremia, cannulation pain related to CT, hematoma or infiltration, bleeding time, aneurysm, and unsuccessful cannulation were considered.

### 2.4. Data Extraction

First, the studies were independently selected by a reviewer (RP). The titles and content of the abstracts were assessed. Then, the second reviewer (AC) carried out the subsequent verification and validation. This selection was made strictly according to the inclusion and exclusion criteria defined in the protocol published elsewhere [23]. All duplicate studies were refused. When the title and abstract were not sufficiently enlightening, a new search was carried out for the full article. After this selection, the full versions of the potentially eligible studies were extracted.

### 2.5. Quality Assessment

To assess the risk of bias in RCT studies, we used the Revised Cochrane Risk-of-Bias Tool for Randomized Trials (RoB 2) tool [24]. According to the number of articles selected and the evidence found, studies with low methodological quality were excluded. Therefore, each study was categorized as presenting low risk, high risk, or unclear risk of bias. For non-randomized studies, the Risk of Bias In Non-Randomized Studies of Interventions (ROBINS-I) [25] was used.

### 2.6. Statistical Analysis

The studies selected for the systematic review were presented in a summary table with the following main attributes: the author’s name, year of publication, country, study design, sample size (*n*), and outcome analysis method. Outcomes: participant’s characteristics (average age, comorbidities), context of dialysis (hospital, clinic, or home), follow-up of the study (months), and primary and secondary outcomes obtained, including the pain scoring tool used to define the severity of pain. We performed a meta-analysis only when studies were sufficiently homogeneous in terms of participants, interventions, outcomes, measurement, and method of aggregation (e.g., mean, proportion). We presented the results in a narrative form when statistical comparisons were not possible. Tables and figures were included to facilitate the presentation of the data. Meta-analysis was performed with the generic inverse variance method using Cochrane Collaboration Review Manager software (RevMan 5.4) for Windows.

## 3. Results

### 3.1. Characteristics of the Included Studies

We selected five RCTs [13,16,19,26,27], one controlled clinical trial [28], and six observational studies [14,29,30,31]. Two were crossover studies [32,33], and the characteristics are shown in Table 1 and Table S1. For the meta-analysis, only RCT studies and outcomes that could be merged were considered.

The studies were published between 2010 and 2022 with the inclusion of 717 patients in the RCT studies and 633 in the observational ones. The studies were mostly carried out in one clinic, with three in multiple clinics and one between a hospital and multicenter. The control group assumed a wide range of designations such as traditional RL [26] (TRL), standard needling [13,16] (SN), usual practice [27] (UP), traditional method [29] (TM), and area technique [31,32]. The remaining studies in the control group used the RL, but among the 12 studies, only 1 used a diagram [19] for guidance of puncture sites during follow-up. The follow-up period varied between 2 and 60 months.

### 3.2. Risk-of-Bias Assessment for RCT Studies

**Figure 2:**
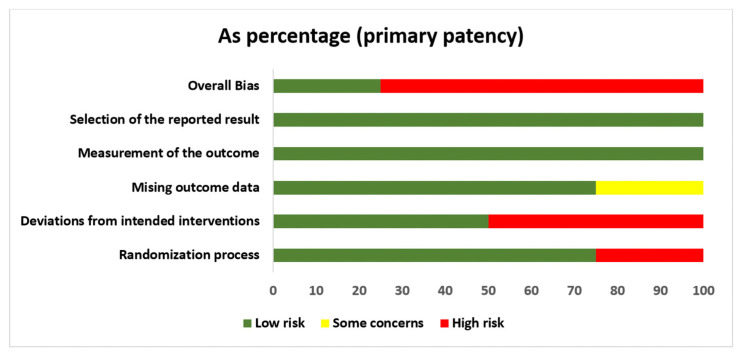
Risk of bias: review author judgments on each risk-of-bias item presented as percentages across the included RCTs.

Figure 2 shows the risk-of-bias assessment that estimated the relative effect of the unassisted primary patency between BH and RL.

Only four studies evaluated this outcome [16,19,27,28] (Figure S1). Risk of bias was observed in the randomization process in one study [28], and no studies blinded personnel or participants due to the visibility and characteristics of the intervention. Some baseline characteristics that may influence the outcome were not evaluated or were significantly different between the two groups. The intended interventions were not illustrated in detail in the trial protocol, mainly cannulation in RL. In two studies, the control group was designated as the usual practice [27], and standard needling [16] and implementation of the CT were not described.

### 3.3. Risk-of-Bias Assessment for Observational Studies

**Figure 3:**
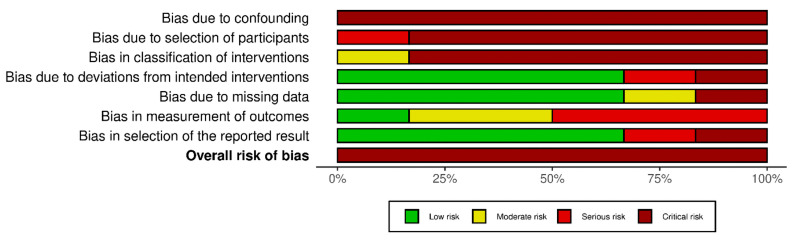
Risk of bias: review author judgments about the risk of bias in non-randomized studies of interventions.

All studies had a critical risk of bias in the first two domains (Figure 3 and Figure S2). No baseline factors that could bias the outcomes were assessed, participant selection was not randomized, and no studies blinded personnel or participants. All involved participants had used AVF before the study began. Some studies had few participants [32,33], and in 50% of the studies, the follow-up was less than four months. Limitations were also identified in the remaining domains; no study described how it implemented the CT in the control group or used a diagram for the RL. Bias was due to missing data [30], and in another study [31], the participants in the control group used RL and area CT.

### 3.4. Primary Outcome—Unassisted Primary Patency

**Figure 4:**
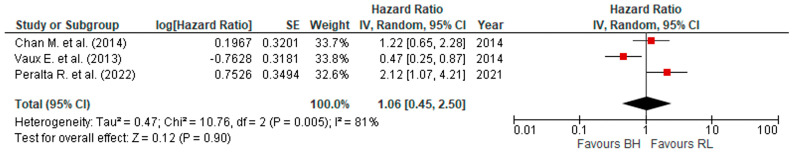
Result of unassisted primary survival rate between BH and RL. The hazard ratio of each study, Chan M. et al. (2014) [28], Vaux E. et al. (2013) [27], and Peralta R. et al. (2022) [19], is shown in red.

Four RCTs reported this outcome, and the results are shown in Table S2. For the meta-analysis, three studies [19,27,28] were used that reported data in a hazard ratio, as shown in Figure 4. The test showed high heterogeneity among the studies, and using the random effects model (*p* < 0.1 e I^2^ = 81%), it was not possible to prove which CT allowed greater unassisted primary patency (HR, 1.06 (95% CI 0.45–2.50) *p* = 0.90).

### 3.5. Number of Interventions in the Fistula

**Figure 5:**
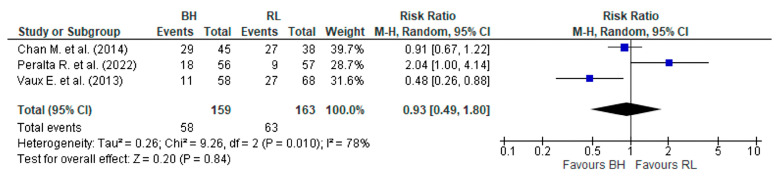
Result of the number of interventions in the AVF between the buttonhole and the rope ladder. The risk ratio of each study, Chan M. et al. (2014) [28], Peralta R. et al. (2022) [19], and Vaux E. et al. (2013) [27], is shown in blue.

There were three studies (Table S3) that reported the number of interventions for AVF and after merging data (Figure 5) showed the existence of high heterogeneity. When using the random effects model (*p* < 0.1 e I^2^ = 78%), it was not possible to prove which CT allowed a lower rate of interventions (angioplasty or surgery) in AVF (RR, 0.93 (95% CI 0.49–1.80) *p* = 0.84).

### 3.6. Arteriovenous Fistula Thrombosis

**Figure 6:**
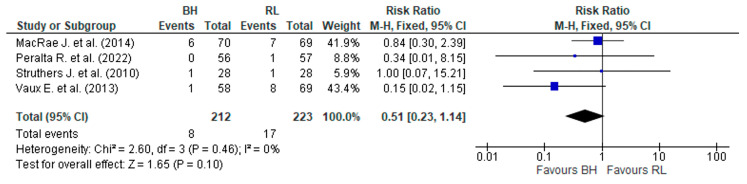
Result of the number of cases of thrombosis in the AVF between the buttonhole and the rope ladder. The risk ratio of each study, MacRae J. et al. (2014) [16], Peralta R. et al. (2022) [19], Struthers J. et al. (2010) [26], and Vaux E. et al. (2013) [27], is shown in blue.

In the four studies that reported AVF thrombosis [16,19,26,27] (Table S3), apparently, there was a higher incidence of thrombosis in the control group. At least one study [27] showed the highest frequency of thrombosis events related to usual CT (eight events) compared to BH (one event). After merging the data (Figure 6) of these four studies, they were shown to be homogeneous using the fixed effects model (*p* > 0.1 e I^2^ = 0%). However, it was not possible to confirm which CT allowed a lower frequency of AVF thrombosis (RR, 0.51 (95% CI 0.23–1.14) *p* = 0.10).

### 3.7. Bacteremia and/or Localized Signs of Infection Related to Vascular Access

All six RCTs (Table S4) assessed the frequency of bacteremia and localized signs of infection related to vascular access. Three studies [13,16,28] reported a higher incidence of bacteremia associated with BH, and one [27] showed more infection rates associated with the usual-practice group (0.09/1000 AVF days).

Vascular access infection was reported by three observational studies [14,30,31] but with different methodologies for assessment and presentation of results. Even so, the studies by van Loon et al. [14] and Glerup R. et al. [31] showed a significantly higher rate of infection (*p* < 0.001) associated with BH (Table S5).

**Figure 7:**
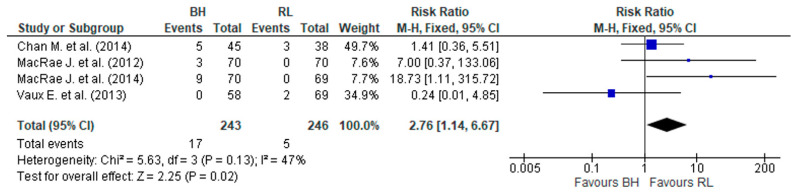
Result of the rate of bacteremia associated with cannulation techniques in arteriovenous fistulas. The risk ratio of each study, Chan M. et al. (2014) [28], MacRae J. et al. (2012) [13], MacRae J. et al. (2014) [16], and Vaux E. et al. (2013) [27], is shown in blue.

RCTs [13,16,27,28] were merged using the fixed effect model. The test showed homogeneity among studies, *p* > 0.1 and I^2^ = 47%. A significant difference was observed (Figure 7) in the incidence of bacteremia associated with BH (RR, 2.76 (95% CI 1.14–6.67) *p* = 0.02).

There also seemed to be a higher rate of localized infection in the BH group. MacRae J. et al. [13] showed a significant difference (*p* = 0.003) in local signs of infection associated with BH (50 per 1000/HD sessions) compared with the SN group (22.4/1000 HD sessions).

### 3.8. Cannulation Pain

There were three RCTs [13,26,27] (Table S6) that assessed cannulation pain and reported it as a median, but these data could not be merged. However, there seemed to be a marginal advantage in the Vaux et al. study [27] (*p* = 0.05) regarding pain reduction in the control group. In this study, eight patients asked to change to the usual practice because of pain associated with the BH. In contrast, three observational studies [29,32,33] (Table S7) showed a significant reduction in pain (*p* = 0.0049, and *p* < 0.001) when using BH. Of the eight studies evaluating cannulation pain, in five studies, the participants used analgesic cream.

### 3.9. Hematoma Associated with Cannulation Techniques

There were two RCTs [13,26] (Table S8) and two observational studies [14,30] (Table S9) that reported a reduction in the rate of hematomas associated with BH. MacRae et al. [13] showed a significant reduction in the rate of hematomas using BH (*p* = 0.003) compared with SN. Van Loon et al. [14] also found that the patients in the BH group had lesser hematoma formation (*p* < 0.0001). Apparently, there was an advantage associated with BH in reducing hematomas, but it was not possible to merge data because the presentation of the results was different. We understand that further studies are needed to assess the frequency of hematomas associated with CT.

### 3.10. Bleeding Time Post-Dialysis

The three RCTs [13,26,27] (Table S8) failed to show the advantage of BH in bleeding time after needle removal. The data could not be merged because of the various definitions of hemostasis and because the data were presented differently. Contrarily, three observational studies [29,32,33] concluded that the bleeding time was significantly lower in patients in the BH group (Table S9).

### 3.11. Aneurysm Formation/Aneurysm Enlargement

There seemed to be unanimity of both RCTs (Table S10) and observational studies (Table S11) in finding that BH was associated with a reduction in aneurysms. The buttonhole significantly reduced existing aneurysm enlargement when it was compared with traditional rope ladder needling [26] and usual practice [27]. When assessing the prospect of aneurysm formation, only one study [19] reported that BH was advantageous over RL. The data could not be merged because of the various definitions of aneurysm enlargement and because the data were presented differently. Observational studies [14,29,30] reported that patients in the BH group had less aneurysm formation, but the follow-up in these studies was short, between 3 and 9 months.

### 3.12. Unsuccessful Cannulation

Nurses perceived significantly higher levels of difficulty with both arterial and venous cannulation in the BH [13] group (*p* < 0.001) compared to standard CT mainly after the fourth month. In another study [27], BH could not be implemented or there were subsequent problems with cannulation in four (6.89%) patients. There were two observational studies [14,30] (Table S11) that assessed unsuccessful cannulations and concluded that BH was associated with a significant increase in miscannulation [14] and where patients in the control group required more than two cannulation attempts [30]. The authors mentioned that miscannulation in the BH group may be attributed to the “trampoline” effect due to the wrong angle of cannulation when the needle is inserted in the tunnel; the needle encounters greater resistance because it does not have the same penetration capacity.

## 4. Discussion

The outcome measures used were numerous and heterogeneous at every level—measurement, metric, method of aggregation, time point of measurement, and follow-up—making it very difficult to reliably evaluate the comparative effectiveness of interventions. There were no attempts to standardize some definitions, mainly the CT used in the control group, which had implications for decisions in clinical practice and improvement in the quality of life of patients under hemodialysis. The different puncture classifications in the control group described above led us to think that they were different puncture methods, or we presumed that the studies used area CT instead of RL. Only one study used patients with incident AVF and described in detail how RL was implemented. They also used the multiple single cannulation technique (MuST) [19,34,35], recently described as a hybrid CT between the rope ladder and the buttonhole that incorporates the benefits from both with promising results. The experimental group and the control group used different CT methods, and therefore blinding could not be applied to patients, nurses, or researchers. Half of the observational studies had fewer than 50 participants, and two were clinical crossover studies with only 21 and 31 patients. Consequently, our comparative assessments of the meta-analysis results from RCTs to guide evidence-based clinical practice are likely to be problematic. The need to standardize outcome measures for vascular access complications has been recognized and several proposals have been published over the last two decades [8,22,36].

Other studies [37] also faced limitations. The outcome measures were assessed in dozens of different ways, and this made it impossible to compare the results across trials and determine that all trials contributed relevant and usable information.

The results of this meta-analysis indicated that BH did not show evidence of superiority in primary patency or in the reduction in the number of interventions when compared with RL. In the study by MacRae et al. [16], no significant differences (*p* = 0.20) were found in AVF survival, even with longer follow-up. These are very important outcome indicators, but there was limited research and the few selected studies revealed considerable heterogeneity (I^2^ = 81%). Therefore, it is recommended that future studies perform more analysis on fistula survival.

The results of this meta-analysis are inconclusive if BH reduces the number of cases of thrombosis; however, there was a clear trend toward a lower incidence of this event. Two previous systematic reviews [38,39] concluded that BH significantly reduced the occurrence of thrombosis. However, in our study, the higher incidence of thrombosis was associated with studies [16,26,27] in which BH was compared with other often ill-defined CTs such as standard needling, traditional rope ladder, and usual practice.

The results indicated that BH has a higher risk of AVF bacteremia than RL. These results are similar to those of another systematic review [40], which showed that infection risk was approximately threefold higher with BH cannulation (RR, 3.34; 95% CI, 0.91 to 12.20; *p* = 0.07). However, we must consider that there were only four trials with a small sample size (243 versus 246) and a small event size (17 versus 5), and these results showed a lack of statistical power. A retrospective observational study [17] using National Healthcare Safety Network (NHSN) surveillance data concluded that BH was associated with a significantly higher risk of access-related bloodstream infection (adjusted relative risk (aRR), 2.6; 95% CI, 2.4–2.8) and local access-site infection (aRR, 1.5; 95% CI, 1.4–1.6) than RL. Despite the re-education programs associated with a strict asepsis policy, audit cycles, and the active eradication of staphylococcus aureus bacteremia, infection rates remained high in BH [18].

However, other studies [38,39] did not find significant differences in the risk of infection between BH and RL. This complication may occur late and not be reported in studies with follow-ups of less than 12 months [16]. To support this inference, an observational [31] study with 60 months of follow-up concluded that bacteremia was significantly higher for buttonhole compared to stepladder/area needling.

This study showed that BH did not reduce the incidence of pain, despite the reduction in injury caused by blunt needles. However, this can be explained by the increase in miscannulation and the “trampoline” effect [14,30] using blunt needles. Two other studies drew the same conclusion [38,39].

There is a clear trend in favor of BH in reducing the rate of hematomas and aneurysm development in RCTs and observational studies. This is in accordance with other studies [38,39] that showed some results, but we should not forget that in these literature reviews, some studies used a CT that is different from RL. On the other hand, the significant increase in aneurysms when using RL, even with an implemented protocol, may be associated with the daily use of area cannulation by professionals [14].

As limitations of this study, we found some constraints on the quality of the included studies, especially the observational studies. The multiple definitions of RL in the control group limited the results, with implications for decisions in clinical practice. Also, the follow-up lengths of the studies were short, and outcomes such as infection and new aneurysms occurred late. Another limitation was the small number of studies and participants, so it would have been useful to conduct a sequential analysis of trials [41,42].

## 5. Conclusions

This meta-analysis demonstrated that BH is significantly associated with higher bacteremia; however, it found no differences in AVF primary patency, number of interventions, or thrombosis. Therefore, BH should be exclusively reserved for home dialysis patients or those with anatomical constraints, as described by some authors [10,43].

To select the best CT for each person, it is necessary to adopt a decision model [44,45] that also involves the patient. In this way, we recommend that VA care should be extended to the patient with education, promoting the development of self-care behaviors by providing the necessary knowledge to patients [46]. To avoid the indiscriminate use of area CT, RL must be implemented with a diagram adjusted to each patient.

## Figures and Tables

**Table 1:** Characteristics of included randomized clinical trial.

First Author	Publication Year	Origin	Study Design	Context	Sample Size	Interventions		Protocol		Follow-Up (Months)	Outcomes
						Experimental Group	Control Group	Experimental Group	Control Group		
MacRae J. et al. [13]	2012	Canada	RCT	Center	140	BH	Standard needling	Specified	Not specified	12	➀ ➁ ➂ ➄
MacRae J. et al. [16]	2014	Canada	RCT	Center	139	BH	Standard needling	Specified	Not specified	19.2 vs. 17.2	➄ ➇ ➈ ➉
Peralta R. et al. [19]	2022	Portugal	RCT	Multicentric	172	MuST	BH and RL	Specified	Specified	12	➃ ➄ ➈ ➉
Struthers J. et al. [26]	2010	United Kingdom	RCT	Center	56	BH	Traditional RL	Specified	Not specified	6	➀ ➁ ➂ ➃ ➄ ➇
Vaux E. et al. [27]	2013	United Kingdom	RCT	Center	127	BH	Usual practice	Specified	Not specified	12	➀ ➁ ➂ ➃ ➄ ➈ ➉
Chan M. et al. [28]	2014	USA	CCT	Center	83	BH	RL	Specified	Not specified	12	➄ ➈

Note: ➀ pain; ➁ hematoma; ➂ bleeding time; ➃ aneurism formation/development; ➄ bacteremia related to vascular access; ➇ thrombosis; ➈ AVF survival; ➉ number of interventions. Abbreviations: RCT: randomized clinical trial; CCT: controlled clinical trial; BH: buttonhole; MuST: multiple single cannulation technique; RL: rope ladder.

## Data Availability

The data presented in this study are available on request from the corresponding author.

## Supplementary Materials

The following supporting information can be downloaded at: https://www.mdpi.com/article/10.3390/jcm12185946/s1, Figure S1: Summary of the risk of bias of the 5 domains assessed in each RCT; Figure S2: Summary of the risk of bias of the 7 domains assessed from the observational studies; Table S1: Characteristics of included observational studies; Table S2: Relative effect of the unassisted primary patency of arteriovenous fistula; Table S3: Number of interventions and thrombosis as the outcome of cannulation techniques in arteriovenous fistulas; Table S4: Bacteremia and local signs of infection as an outcome of the cannulation technique in arteriovenous fistulas in RCT studies; Table S5: Bacteremia and local signs of infection as an outcome of the cannulation technique in arteriovenous fistulas in observational studies; Table S6: Pain as an outcome of the cannulation technique in arteriovenous fistulas in RCT studies; Table S7: Pain as an outcome of the cannulation technique in arteriovenous fistulas in observational studies; Table S8: Hematoma and bleeding time as an outcome of the cannulation technique in arteriovenous fistulas in RCT studies; Table S9: Hematoma and bleeding time as an outcome of the cannulation technique in arteriovenous fistulas in observational studies; Table S10: Aneurism formation/development as an outcome of the cannulation technique in arteriovenous fistulas in RCT studies; Table S11: Aneurism formation/development and unsuccessful cannulation as an outcome of the cannulation technique in arteriovenous fistulas in observational studies.
