# Supplementary material for: Cannulation Technique of Vascular Access in Hemodialysis and the Impact on the Arteriovenous Fistula Survival: Systematic Review and Meta-Analysis
Source: J Clin Med. 2023 Sep 13;12(18):5946. doi: 10.3390/jcm12185946 (PMC10532371; doi:10.3390/jcm12185946)
Supplement: Supplementary file 1 [file jcm-12-05946-s001.zip › Table S1 - Characteristics of observational studies.pdf]

Table S1. Characteristics of included observational studies

First author	Publication year	Origin	Study design	Context	Sample size	Interventions		Protocol		Follow up (months)	Outcomes
						Experimental group	Control group	Experimental group	Control group		
van Loon et al. [14]	2010	Netherlands	Observational	Multicentric	145	BH	RL	Specified	Specified	9	① ② ④ ⑤ ⑥ ⑦
Pergolotti et al. [29]	2011	USA	Observational	Center	45	BH	Traditional method	Not specified	Not specified	4	① ③ ④
*Sukthinthai et al. [32]	2012	Thailand	Cross-over study	Center	21	BH	Area puncture method	Specified	area puncture	11 (3 phases)	① ③
Mi-Kyoung K. et al. [33]	2013	South Korea	Cross-over study	Center	32	BH with Two pairs of tunnel tract	RL	Specified	Not specified	BH 3; RL 2	① ③
Smyth W. et al. [30]	2013	Australia	Observational	Multicentric (hospital & center)	104	BH	RL	Specified	Not specified	3	① ② ④ ⑤ ⑥
Glerup R. et al. [31]	2019	Denmark	Observational	Multicentric	286	BH	Stepladder or area puncture	Not specified & Self-cannulation	Not specified & Self-cannulation	60	⑤

Note: ① pain; ② hematoma; ③ bleeding time; ④ aneurisms formation/development; ⑤ bacteremia related to vascular access; ⑥ unsuccessful cannulation; ⑦ maintenance of functionality; ⑧ thrombosis; ⑨ AVF survival; ⑩ number of interventions. Abbreviations: TC: Cannulation technique; BH: Buttonhole; MuST: Multiple single cannulation technique. RL: Rope-ladder

* The study was conducted over 11 months in 3 phases but does not specify the duration of each one.
