# Supplementary material for: Cannulation Technique of Vascular Access in Hemodialysis and the Impact on the Arteriovenous Fistula Survival: Systematic Review and Meta-Analysis
Source: J Clin Med. 2023 Sep 13;12(18):5946. doi: 10.3390/jcm12185946 (PMC10532371; doi:10.3390/jcm12185946)
Supplement: Supplementary file 1 [file jcm-12-05946-s001.zip › Table S10 - Aneurism formationdevelopment as an outcome of the cannulation technique in arteriovenous fistulas in RCT studies.pdf]

**Table S10**. Aneurism formation/development as an outcome of the cannulation technique in arteriovenous fistulas in RCT studies

| First author | Study design | Average age (years) | Sample size | | Outcome | | *P* Value | Notes |
|---|---|---|---|---|---|---|---|---|
| | | | Experimental group | Control group | Experimental group | Control group | | |
| **Aneurism formation/development** | | | | | | | | |
| Struthers J. et al. (2010) [26] | RCT | BH = 61<br>TRL = 60 | 28 | 28 | Mean 1%<br>SEM ± 22% | Mean 30%<br>SEM ± 7% | < 0,01 | Assessed average increase in size |
| Vaux E. et al. (2013) [27] | RCT | BH = 62<br>UP = 64 | 58 | 69 | 23%<br>2 (4%) | 67%<br>9 (17%) | -- | - Existing aneurysm enlargement<br>- New aneurysm formation |
| Peralta R. et al. (2022) [19] | RCT | MuST = 68.48 | 59 | | n = 1 (1.7%) | | | Assessed new aneurism formation |
| | | BH = 68.78 | | 56 | | 0 | | |
| | | RL = 65.95 | | 57 | | n = 1 (1.8%) | -- | |

Abbreviations: RCT: randomized clinical trial; BH: buttonhole; TRL: traditional RL; UP: usual practice; MuST: multiple single cannulation technique; RL: rope-ladder SEM: standard error of the mean
