# Supplementary material for: Cannulation Technique of Vascular Access in Hemodialysis and the Impact on the Arteriovenous Fistula Survival: Systematic Review and Meta-Analysis
Source: J Clin Med. 2023 Sep 13;12(18):5946. doi: 10.3390/jcm12185946 (PMC10532371; doi:10.3390/jcm12185946)
Supplement: Supplementary file 1 [file jcm-12-05946-s001.zip › Table S11 - Aneurism formationdevelopment and Unsuccessful cannulation as an outcome of the cannulation technique in arteriovenous fistulas in Observ.pdf]

Table S11. Aneurism formation/development and Unsuccessful cannulation as an outcome of the cannulation technique in arteriovenous fistulas in Observational studies

First author	Study design	Average age (years)	Sample size		Outcome		*P* Value	Notes
			Experimental group	Control group	Experimental group	Control group		
**Aneurism formation/development**								
van Loon et al. (2010) [14]	Observational	BH = 67<br>RL = 65	75	70	n =1 (1%)	n =47 (67%)	< 0.001	Assessed aneurysm formation
Pergolotti et al. (2011) [29]	Observational	BH = 56<br>TRL = 66.5	21	24	20%	45.8%	-	Assessed aneurysm sized at 1 mm or more in elevation.
Smyth W et al. (2013) [30]	Observational	BH = 60<br>RL =61	41	63	3 (7.3%)	18 (28.6%)	= 0.017	Assessed aneurysm formation
**Unsuccessful cannulation**								
van Loon et al. (2010) [14]	Observational	BH = 67<br>RL = 65	75	70	Mean = 8.1 (SD =7)	Mean = 3.7 (SD = 4.7)	< 0.001	The mean (SD) number of miscannulation
Smyth W et al. (2013) [30]	Observational	BH = 60<br>RL = 61	41	63	n = 92 (6.7%)	68 (3.6%)	< 0.0001	The number of unsuccessful cannulation attempts

Abbreviations: BH: buttonhole; TRL: traditional RL; RL: rope-ladder; SD: standard deviation
