# Supplementary material for: Cannulation Technique of Vascular Access in Hemodialysis and the Impact on the Arteriovenous Fistula Survival: Systematic Review and Meta-Analysis
Source: J Clin Med. 2023 Sep 13;12(18):5946. doi: 10.3390/jcm12185946 (PMC10532371; doi:10.3390/jcm12185946)
Supplement: Supplementary file 1 [file jcm-12-05946-s001.zip › Table S2 - Relative effect of the unassisted primary patency of arteriovenous fistula.pdf]

Table S2. Relative effect of the unassisted primary patency of arteriovenous fistula

First author	Study design	Average age (years)	Sample size		Outcome		*P* Value log-rank test	Notes
			Experimental group	Control group	Experimental group	Control group		
Vaux E. et al. (2013) [27]	RCT	BH = 62	58	69	100%	86%	= 0.005	- AVF survival at 1 year – (assisted primary patency)
		UP = 64			74%	49%	= 0.01	- Primary patency at 1-year (intent to treat)<br>- HR 0.46 (95% CI 0.25-0.87) *p* = 0.02
MacRae J. et al. (2014) [16]	RCT	BH =70.3<br>SN = 66.7	70	69	Median 18.4 (IQR, 10.9 - 32.7)	Median 16 (IQR; 10.6 - 29.3)	= 0.2	- Median access survival (months)
					Median 12.7 (IQR, 7.8 - 19.2)	Median 16 (IQR, 10.6 - 29.3)	= 0.2	- Median access survival (intent to treat) months
Chan M. et al. (2014) [28]	CCT	BH = 60.9<br>RL = 64.1	45	38	57%	60%	= 0.50	- Primary patency at 1-year<br>- HR 1.22 (95% CI 0.65-2.28) *p* = 0.53
* Peralta R. et al. (2022) [19]	RCT	MuST= 68.48	59		76.6%			- Primary patency at 1-year
		BH = 68.78		56		59.6%	= 0.003	- HR 2.13 (95% CI 1.07-4.21) *p* = 0.03
		RL = 65.95		57		76.8%		

Abbreviations: RCT: randomized clinical trial; CCT: controlled clinical trial; BH: Buttonhole; UP: usual practice; SN: standard needling; MuST: multiple single cannulation technique; RL: rope-ladder; IQR: interquartile range; HR: hazard ratio.

* Study compares three cannulation techniques: MuST was experimental group versus buttonhole versus rope-ladder.
