# Supplementary material for: Cannulation Technique of Vascular Access in Hemodialysis and the Impact on the Arteriovenous Fistula Survival: Systematic Review and Meta-Analysis
Source: J Clin Med. 2023 Sep 13;12(18):5946. doi: 10.3390/jcm12185946 (PMC10532371; doi:10.3390/jcm12185946)
Supplement: Supplementary file 1 [file jcm-12-05946-s001.zip › Table S3 - Number of interventions and thrombosis as the outcome of cannulation techniques in arteriovenous fistulas.pdf]

Table S3. Number of interventions and thrombosis as the outcome of cannulation techniques in arteriovenous fistulas

First author	Study design	Average age (years)	Sample size		Outcome		*P* Value log-rank test	Notes
			Experimental group	Control group	Experimental group	Control group		
**Number of interventions**								
Vaux E. et al. (2013) [27]	RCT	BH = 62<br>UP = 64	58	69	n = 11 (19%)	n =27 (39%)		
Chan M. et al. (2014) [28]	CCT	BH = 60.9<br>RL = 64.1	45	38	n = 29 (64%)	n =27 (71%)	= 0.52	
Peralta R. et al. (2022) [19]	RCT	BH = 68.78<br>RL = 65.95	56	57	n = 18 (31.6%)	n = 9 (16.1%)		
**Thrombosis**								
Struthers J. et al. (2010) [26]	RCT	BH = 61<br>SRL= 60	28	28	1	1		
Vaux E. et al. (2013) [27]	RCT	BH = 62<br>UP = 64	58	69	1	8		BH - 1 AVF Successful thrombectomy<br>UP - 2 AVF Successful thrombectomy
MacRae J. et al. (2014) [16]	RCT	BH = 70.3<br>SN = 66.7	70	69	n =6 (8.6%)	n =7 (10.1%)	= 0.6	
Peralta R. et al. (2022) [19]	RCT	BH = 68.78<br>RL = 65.95	56	57	0	1		

Abbreviations: RCT: randomized clinical trial; CCT: controlled clinical trial; BH: Buttonhole; UP: usual practice; SN: standard needling; MuST: multiple single cannulation technique; RL: rope-ladder.
