# Supplementary material for: Cannulation Technique of Vascular Access in Hemodialysis and the Impact on the Arteriovenous Fistula Survival: Systematic Review and Meta-Analysis
Source: J Clin Med. 2023 Sep 13;12(18):5946. doi: 10.3390/jcm12185946 (PMC10532371; doi:10.3390/jcm12185946)
Supplement: Supplementary file 1 [file jcm-12-05946-s001.zip › Table S4 - Bacteremia and local signs of infection as an outcome of the cannulation technique in arteriovenous fistulas in RCT studies.pdf]

Table S4. Bacteremia and local signs of infection as an outcome of the cannulation technique in arteriovenous fistulas in RCT studies

First author	Study design	Average age (years)	Sample size		Outcome		*P* Value	Notes
			Experimental group	Control group	Experimental group	Control group		
**Bacteremia and local signs of infection related to access (n = events)**								
Struthers J. et al. (2010) [26]	RCT	BH = 61<br>TRL = 60	28	28	1	0	--	- 1 local infection, had antibiotic therapy
MacRae J. et al. (2012) [13]	RCT	BH = 70.3<br>SN = 66.7	70	70	50/1000 HD sessions<br>3	22.4/1000 HD sessions<br>0	= 0.003	- Rate of localized signs of infection<br>- BH - 3 S. aureus bacteremia and 9 Site abscesses that had antibiotic therapy
Vaux E. et al. (2013) [27]	RCT	BH = 62<br>UP = 64	58	69	0.12/1000 AVF days<br>0	0<br>0.09/1000 AVF days	--	BH 2 exit-site infections<br>UP = 2 Bacteremia.
MacRae J. et al. (2014) [16]	RCT	BH = 70.3<br>SN = 66.7	70	69	3<br>9	0<br>0	< 0.001	- BH – 3 Localized/exit site<br>- BH 9 S. aureus bacteremia<br>- Median time to first infection for buttonhole was 11.1 months
Chan M. et al. (2014) [28]	CCT	BH = 60.9<br>RL = 64.1	45	38	5 (11%)	3 (8%)	0.62	- Bacteriemia.
Peralta R. et al. (2022) [19]	RCT	MuST= 68.48<br>BH = 68.78<br>RL = 65.95	59	 56<br>57	0	 1 (1.8%)<br>0	--	 - Localized signs of infection

Abbreviations: RCT: randomized clinical trial; CCT: controlled clinical trial; BH: Buttonhole; TRL: traditional RL; UP: usual practice; SN: standard needling; MuST: multiple single cannulation technique; RL: rope-ladder; HD: hemodialysis
