# Supplementary material for: Cannulation Technique of Vascular Access in Hemodialysis and the Impact on the Arteriovenous Fistula Survival: Systematic Review and Meta-Analysis
Source: J Clin Med. 2023 Sep 13;12(18):5946. doi: 10.3390/jcm12185946 (PMC10532371; doi:10.3390/jcm12185946)
Supplement: Supplementary file 1 [file jcm-12-05946-s001.zip › Table S5 - Bacteremia and local signs of infection as an outcome of the cannulation technique in arteriovenous fistulas in Observational studies.pdf]

Table S5. Bacteremia and local signs of infection as an outcome of the cannulation technique in arteriovenous fistulas in Observational studies

First author	Study design	Average age (years)	Sample size		Outcome		*P* Value	Notes
			Experimental group	Control group	Experimental group	Control group		
**Bacteremia and local signs of infection related to access (n = events)**								
van Loon et al. (2010) [14]	Observational	BH = 67<br>RL = 65	75	70	2<br>2	0<br>0		- BH 2 Two local infections<br>- BH 2 Staphylococcus aureus
Smyth W et al. (2013) [30]	Observational	BH = 60<br>RL = 61	41	63	2.34/1000 HD sessions	6.38/1000 HD sessions	=1.0	- Clinical signs of infection: BH = 3 (7.3%) vs RL = 4 (6.3%)
Glerup R et al. (2019) [31]	Observational	BH = 63.7<br>Steplader/area =71.2	144	142	0.268/1000 Fistula-days	0.038/1000 Fistula-days	< 0.001	Staphylococcus aureus bacteremia:<br>BH = 43 (29.86%)<br>RL/área = 5 (3.52%)

Abbreviations: BH: buttonhole; RL: rope-ladder; HD: hemodialysis
