# Supplementary material for: Cannulation Technique of Vascular Access in Hemodialysis and the Impact on the Arteriovenous Fistula Survival: Systematic Review and Meta-Analysis
Source: J Clin Med. 2023 Sep 13;12(18):5946. doi: 10.3390/jcm12185946 (PMC10532371; doi:10.3390/jcm12185946)
Supplement: Supplementary file 1 [file jcm-12-05946-s001.zip › Table S6 - Pain as an outcome of the cannulation technique in arteriovenous fistulas in RCT studies.pdf]

Table S6. Pain as an outcome of the cannulation technique in arteriovenous fistulas in RCT studies

First author	Study design	Average age (years)	Sample size		Outcome		*P* Value	Notes
			Experimental group	Control group	Experimental group	Control group		
Struthers J. et al. (2010) [26]	RCT	BH = 61<br>TRL = 60	28	28	median 2.5	median 1		Was used visual analogue scoring system. Used local anesthetic. Nine of 22 patients in the BH reduced or stopped local anesthetic.
MacRae J. et al. (2012) [13]	RCT	BH = 70.3<br>SN = 66.7	70	70	median 1.5<br>IQR, 0.5 – 3.4	median 1.2<br>IQR, 0.4 – 2.4	= 0.57	Was used visual analogue scale. One-quarter of them used anesthetic with needling.
Vaux E. et al. (2013) [27]	RCT	BH = 62<br>UP = 64	58	69	median 1.3<br>IQR, 1.2 -1.9	median 1.2<br>IQR, 1.0 -1.5	= 0.05	Was used self-reported modified numeric pain rating scale. Used local anesthetic. 8 patients in the BH abandoned cannulation due to pain.

Abbreviations: RCT: randomized clinical trial; BH: Buttonhole; TRL: traditional RL; UP: usual practice; SN: standard needling; RL: rope-ladder; IQR: interquartile range
