# Supplementary material for: Cannulation Technique of Vascular Access in Hemodialysis and the Impact on the Arteriovenous Fistula Survival: Systematic Review and Meta-Analysis
Source: J Clin Med. 2023 Sep 13;12(18):5946. doi: 10.3390/jcm12185946 (PMC10532371; doi:10.3390/jcm12185946)
Supplement: Supplementary file 1 [file jcm-12-05946-s001.zip › Table S7 - Pain as an outcome of the cannulation technique in arteriovenous fistulas in Observational studies.pdf]

Table S7. Pain as an outcome of the cannulation technique in arteriovenous fistulas in Observational studies

First author	Study design	Average age (years)	Sample size		Outcome		*P* Value	Notes
			Experimental group	Control group	Experimental group	Control group		
van Loon et al. (2010) [14]	Observational	BH = 67<br>RL = 65	75	70	Mean 1.6 (0-5)	Mean 1.0 (0 – 5.4)	< 0.0001	Was used Verbal Rating Scale. Used local anaesthetic cream. Use of local anaesthetic cream (BH 8% vs RL 30% $p < 0.0001$)
Pergolotti et al. (2011) [29]	Observational	BH = 56<br>TRL = 66.5	21	24	Average pain 47.8% or less	-	= 0.0049	Was used Visual Analog Scale. Used analgesic medications.
SuKthinthai et al. (2012) [32]	Cross-over	56.3	21	21	V = 1.38 ± 0.75<br>A =1.20 ±0.90	V = 5.88 ±0.82<br>A = 6.03 ±0.90	< 0.0001<br>< 0.0001	Was used Verbal Rating Scale.
Mi-Kyoung KIM et al. (2013) [33]	Cross-over	62.4	32	32	V = 5.1 ±1.8<br>A = 3.3 ±1.8	V = 6.3 ±1.3<br>A = 6.1 ±1.2	< 0.001<br>< 0.001	Was used Visual Analog Scale.
Smyth W et al. (2013) [30]	Observational	BH = 60<br>RL = 61	41	63	1.9 (1 - 6)<br>SD =1.069	1.82 (1-5)<br>SD = 0.934	= 0.607	Was used Visual Analog Scale. Local anaesthetic is used.

Abbreviations: BH: Buttonhole; TRL: traditional RL; RL: rope-ladder; A: arterial; V: venous; SD: standard deviation.
