# Supplementary material for: Cannulation Technique of Vascular Access in Hemodialysis and the Impact on the Arteriovenous Fistula Survival: Systematic Review and Meta-Analysis
Source: J Clin Med. 2023 Sep 13;12(18):5946. doi: 10.3390/jcm12185946 (PMC10532371; doi:10.3390/jcm12185946)
Supplement: Supplementary file 1 [file jcm-12-05946-s001.zip › Table S8 - Hematoma and bleeding time as an outcome of the cannulation technique in arteriovenous fistulas in RCT studies.pdf]

Table S8. Hematoma and bleeding time as an outcome of the cannulation technique in arteriovenous fistulas in RCT studies

First author	Study design	Average age (years)	Sample size		Outcome		*P* Value	Notes
			Experimental group	Control group	Experimental group	Control group		
**Hematoma (n = events)**								
Struthers J. et al. (2010) [26]	RCT	BH = 61<br>TRL = 60	28	28	19	27	--	N/A
MacRae J. et al. (2012) [13]	RCT	BH = 70.3<br>SN = 66.7	70	70	295/1000<br>HD sessions	436/1000<br>HD sessions	= 0.003	Hematoma was categorized based on size
**Bleeding time after needle removal, (min)**								
Struthers J. et al. (2010) [26]	RCT	BH = 61<br>TRL = 60	28	28	Mean 300 ± 26 seconds	Mean 400 ± 31 seconds	SSE	Bleeding time after needle removal
MacRae J. et al. (2012) [13]	RCT	BH=70.3<br>SN = 66.7	70	70	Any bleeding<br>97.2/1000<br>HD Sessions	Any bleeding<br>97.6/1000<br>HD Sessions	= 0.40	no bleeding <5 minutes, 5 –10 minutes, or > 10 minutes bleeding post-HD
Vaux E. et al. (2013) [27]	RCT	BH = 62<br>UP = 64	58	69	median 7.9<br>IQR (6.3 – 10.4)	median 9.1<br>IQR (6.9 -11.3)	= 0.30	Bleeding times in minutes

Abbreviations: RCT: randomized clinical trial; BH: Buttonhole; TRL: traditional RL; UP: usual practice; SN: standard needling; RL: rope-ladder; HD: hemodialysis; IQR: interquartile range
