# Supplementary material for: Cannulation Technique of Vascular Access in Hemodialysis and the Impact on the Arteriovenous Fistula Survival: Systematic Review and Meta-Analysis
Source: J Clin Med. 2023 Sep 13;12(18):5946. doi: 10.3390/jcm12185946 (PMC10532371; doi:10.3390/jcm12185946)
Supplement: Supplementary file 1 [file jcm-12-05946-s001.zip › Table S9 - Hematoma and bleeding time as an outcome of the cannulation technique in arteriovenous fistulas in Observational studies.pdf]

Table S9. Hematoma and bleeding time as an outcome of the cannulation technique in arteriovenous fistulas in Observational studies

First author	Study design	Average age (years)	Sample size		Outcome		*P* Value	Notes
			Experimental group	Control group	Experimental group	Control group		
**Hematoma (n)**								
van Loon et al. (2010) [14]	Observational	BH = 67<br>RL = 65	75	70	Mean 2.0 SD = 3.7	Mean 14 SD = 15.6	< 0.0001	Hematoma: An abnormal localized infiltration of blood caused by needle cannulation
Smyth W et al. (2013) [30]	Observational	BH = 60<br>RL = 61	41	63	n = 11 (26.8%)	n = 20 (31.7%)	= 0.752	
**Bleeding time after needle removal, (min)**								
Pergolotti et al. (2011) [29]	Observational	BH = 56<br>TRL = 66.5	21	24	Hemostasis time less 23.7%	-	= 0.0066	
Sukthinthai et al. (2012) [32]	Cross-over	56.3	21	21	A = 4.19 ± 1.66<br>V = 3.92 ± 1.37	A = 9.12 ± 2.36<br>V = 3.12 ± 2.36	< 0.0001<br>< 0.0001	Hemostasis after needle withdrawal in minutes
Mi-Kyoung KIM et al. (2013) [33]	Cross-over	62.4	32	32	<15 n = 15 (46.9%)<br>16-30 n = 17 (53.1%)<br>-	<15 n = 7 (21.9%)<br>16-30 n = 16 (50%)<br>>30 n = 9 (28.1%)	< 0.001<br>= 0.028	Results referring to the first assessment for A and V cannulation in minutes

Abbreviations: BH: Buttonhole; TRL: traditional RL; RL: rope-ladder; HD: hemodialysis; A: arterial; V: venous; SD: standard deviation
