# Supplementary figures and images for: Cannulation Technique of Vascular Access in Hemodialysis and the Impact on the Arteriovenous Fistula Survival: Systematic Review and Meta-Analysis
Source: J Clin Med. 2023 Sep 13;12(18):5946. doi: 10.3390/jcm12185946 (PMC10532371; doi:10.3390/jcm12185946)

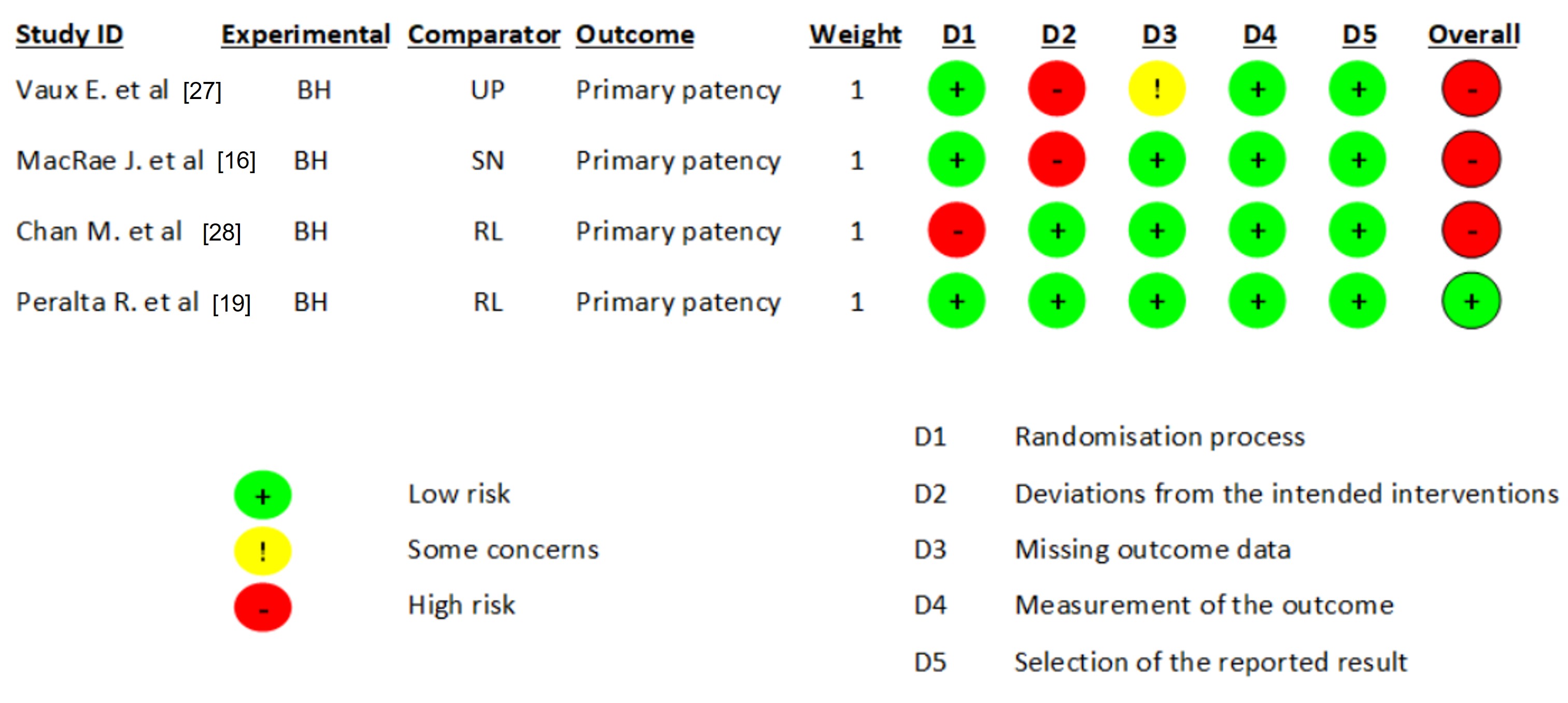

Supplement: Supplementary file 1 [file jcm-12-05946-s001.zip › Figure S1. Summary of the risk of bias of the 5 domains assessed in each RCT.jpg]

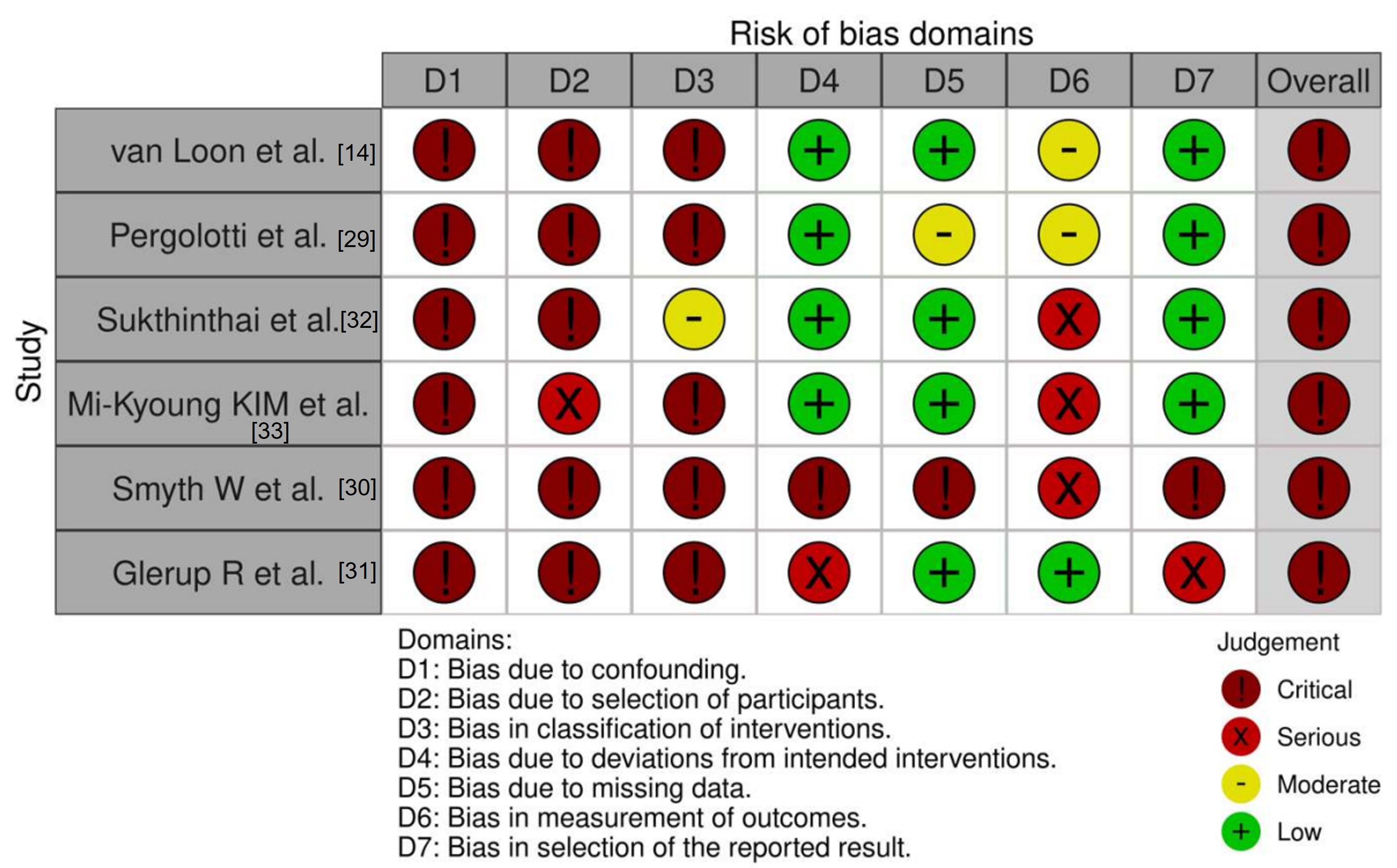

Supplement: Supplementary file 1 [file jcm-12-05946-s001.zip › Figure S2. Summary of the risk of bias of the 7 domains assessed from the observational.jpg]
